# Identification and Quantification of Necroptosis Landscape on Therapy and Prognosis in Kidney Renal Clear Cell Carcinoma

**DOI:** 10.3389/fgene.2022.832046

**Published:** 2022-02-14

**Authors:** Sheng Xin, Jiaquan Mao, Chen Duan, Jiaxin Wang, Yuchao Lu, Jun Yang, Jia Hu, Xiaming Liu, Wei Guan, Tao Wang, Shaogang Wang, Jihong Liu, Wen Song, Xiaodong Song

**Affiliations:** Department of Urology, Tongji Hospital, Tongji Medical College, Huazhong University of Science and Technology, Wuhan, China

**Keywords:** kidney renal clear cell carcinoma, necroptosis, tumor immune microenvironment, prognostic signature, nomogram, bioinformatics

## Abstract

Kidney renal clear cell carcinoma (KIRC) has high morbidity and gradually increased in recent years, and the rate of progression once relapsed is high. At present, owing to lack of effective prognosis predicted markers and post-recurrence drug selection guidelines, the prognosis of KIRC patients is greatly affected. Necroptosis is a regulated form of cell necrosis in a way that is independent of caspase. Induced necroptosis is considered an effective strategy in chemotherapy and targeted drugs, and it can also be used to improve the efficacy of immunotherapy. Herein, we quantified the necroptosis landscape of KIRC patients from The Cancer Genome Atlas (TCGA) database and divided them into two distinct necroptosis-related patterns (C1 and C2) through the non-negative matrix factorization (NMF) algorithm. Multi-analysis revealed the differences in clinicopathological characteristics and tumor immune microenvironment (TIME). Then, we constructed the NRG prognosis signature (NRGscore), which contained 10 NRGs (PLK1, APP, TNFRSF21, CXCL8, MYCN, TNFRSF1A, TRAF2, HSP90AA1, STUB1, and FLT3). We confirmed that NRGscore could be used as an independent prognostic marker for KIRC patients and performed excellent stability and accuracy. A nomogram model was also established to provide a more beneficial prognostic indicator for the clinic. We found that NRGscore was significantly correlated with clinicopathological characteristics, TIME, and tumor mutation burden (TMB) of KIRC patients. Moreover, NRGscore had effective guiding significance for immunotherapy, chemotherapy, and targeted drugs.

## Introduction

Kidney cancer is the third largest malignant tumor in the genitourinary system, with growing morbidity and mortality in recent years. It is estimated that in 2018, >400,000 new cases were diagnosed and >175,000 people died of this disease (Bray et al., 2018). About 90% of kidney cancer was renal cell carcinoma (RCC), 70% of which was kidney renal clear cell carcinoma (KIRC) (Atkins and Tannir, 2018). About 30% of patients have metastasis by the time they are diagnosed. With advances in RCC pathologic staging, the 5-year disease-specific survival (DSS) rate has been reduced by about 10%; however, the median overall survival (OS) for advanced RCC is just 10–15 months (Cairns, 2011). Medication (immunotherapy, chemotherapy, and targeted drugs) is the preferred treatment approach for patients with end-stage or recurrent KIRC. However, due to secondary effects of drugs, individual differences in drug sensitivity, and lack of reliable prognostic biomarkers, there is usually little improvement in the median OS probability after the first round of therapy (Cairns, 2011; Luo et al., 2019). The tumor microenvironment (TME) is closely related to tumor progression and efficacy of immunotherapy and chemotherapy (Wu and Dai, 2017; Hinshaw and Shevde, 2019; Newton et al., 2019). Altering the TME has been a potential strategy for improving the efficacy of anticancer treatments and clinical outcomes.

Necroptosis is a regulated form of cell necrosis in a way that is independent of caspase (Gong et al., 2019). RIPK1 and RIPK3 are upstream molecules of necroptosis, which form oligomeric complexes of necrotic bodies and cause rapid membrane permeability of necrotic cells through MLKL (Galluzzi et al., 2012; Cai et al., 2014). Therefore, necroptosis shows morphological characteristics of cell membrane rupture, gradually translucent cytoplasm, and organelle swelling (Vandenabeele et al., 2010; Chan, 2012). In addition, the release of cell contents leads to exposure of damage-associated molecular patterns (DAMPs) and a strong inflammatory response (Pasparakis and Vandenabeele, 2015). In apoptosis, DAMPs are mostly solidified, so necroptosis is significantly diverse from apoptosis not only in morphology but also in immunology (Kaczmarek et al., 2013). In multiple tumors, the key molecular expression of the necroptosis signaling pathway was reduced, which was related to poor prognosis and enhanced tumor progression and metastasis (Park et al., 2009; Ke et al., 2013; Wang et al., 2013; Feng et al., 2015; Ertao et al., 2016; Stoll et al., 2017). Inducing necroptosis is considered an effective strategy to solve the problem of apoptosis resistance in the process of chemotherapy, and a variety of anticancer drugs have been developed to induce necroptosis (Gong et al., 2019). Furthermore, necroptosis induces NF-κB–derived signals, activates dendritic cells (DCs), increases antigen presentation, and enhances CD8 + T cell–mediated tumor clearance (Snyder et al., 2019). Bioinformatics analysis suggests that RIPK1, RIPK3, and MLKL are associated with T cell dysfunction, and their overexpression predicts prolonged survival in many clinical studies of immune checkpoint inhibitors (ICIs) (Tang et al., 2020). Several animal experiments have explored the synergy of necroptosis in ICIs to produce novel immunotherapy strategies (Kang et al., 2018; Van Hoecke et al., 2018; Snyder et al., 2019). These suggest that necroptosis has great potential in providing effective drug therapy for advanced KIRC patients.

To address the abovementioned point, we clustered 526 KIRC patients from The Cancer Genome Atlas (TCGA) database on the basis of the expression patterns of necroptosis-related genes (NRGs). The differences between necroptosis-related patterns were analyzed in multi-omics analysis, including survival analysis, clinical relevance, tumor immune microenvironment (TIME), and so on. A prognostic signature (NRGscore) that could be used to predict the OS of KIRC patients was then constructed, confirming that it was an independent prognostic indicator. Moreover, a nomogram model was constructed with NRGscore and several clinicopathological characteristics to provide accurate prognosis predictions for clinical patients. Eventually, we have verified that NRGscore was significantly correlated with TIME, somatic mutation, and immunotherapeutic and chemotherapeutic efficacy in KIRC patients.

## Materials and Methods

### Retrieval of Necroptosis-Related Genes

We first obtained eight NRGs from the GOBP_NECROPTOTIC_SIGNALING_PATHWAY gene set in the Molecular Signatures database (MSigDB) (http://www.gsea-msigdb.org/gsea/msigdb/index.jsp). After screening a large number of previous research literature on necroptosis, a necroptosis gene set containing 74 NRGs was finally retrieved (Supplementary Table S1).

### Acquisition and Process of Original Data

Transcription RNA sequencing, clinical information, and somatic mutation of TCGA-KIRC cohort were publicly available in TCGA database (https://portal.gdc.cancer.gov/). Transcription RNA sequencing consisted of 539 KIRC tumor tissues and 72 surrounding normal tissues. It was downloaded as fragments per kilobase of transcript per million mapped reads (FPKM), and gene expression was annotated in an average when an individual gene symbol contained more than one Ensembl ID. After removing the samples without complete OS information, 526 patients were incorporated into the training set. 328 TCGA samples included in the study had somatic mutation information. The E-MATB-1980 dataset (https://www.ebi.ac.uk/arrayexpress/experiments/E-MTAB-1980/) provided the RNA-seq data and clinical information of 101 KIRC samples to be an external test set. All sequencing data were processed with log2 transformation and eliminated batch effects between cohorts before establishing and verifying the prognostic signature through the “sva” package in R.

### Non-Negative Matrix Factorization Clustering

We integrated the RNA-seq data and overall survival (OS) information of TCGA-KIRC and gained the prognosis-related NRGs through univariate COX regression analysis (*p* < 0.05). Non-negative matrix factorization (NMF) was applied to determine distinct necroptosis-related patterns with the help of the “NMF” R package. The NMF algorithm divided the original matrix into two non-negative matrices to identify the potential features in the gene expression profile (Brunet et al., 2004). The deposition was repeated and the results were aggregated to obtain consistent clustering. According to the cophenetic coefficient, contour, and sample size, k = 2 was determined as the best cluster number. All the prognosis-related NRGs were selected to construct a principal component analysis (PCA) scoring system with the “prcomp” function in R.

### Gene Set Enrichment Analysis

GSEA is a nonparametric and unsupervised algorithm that transforms an isolate gene expression matrix to an expression matrix of particular gene sets as features. The algorithm is implemented based on the “clusterProfiler,” “enrichplot,” and “DOSE” R packages. We downloaded the gene sets of “c2. cp.kegg.v7.4. symbols,” “h.all.v7.4. symbols,” “c2. cp.reactome.v7.4. symbols,” “c2. cp.biocarta.v7.4. symbols,” and “c2. cp.pid.v7.4. symbols” from the MSigDB database for GSEA. The statistical differences of the expression matrix after transformation were analyzed by the “limma” package.

### Evaluation of the Tumor Immune Microenvironment

Single-sample gene set enrichment analysis (ssGSEA), ESTIMATE, and CIBERSORT were used in R to assess the TIME status of each KIRC sample. ssGSEA investigated congenital and adaptive immune cells as well as a variety of immune-related functions. The normalized enrichment score (NES) was to embody the relative amount of each TIME infiltration unit in patients. ESTIMATE predicted the level of infiltrating matrix and immune cells by calculating stromal and immune scores and comprehensively obtained the ESTIMATE score for evaluating the TIME. We also assessed the relative fraction of 22 tumor-infiltrating immune cells (TIICs) in each cancer sample with the CIBERSORT algorithm. *P* < 0.05 was the threshold of a credible sample for estimating the proportion of immune cells.

### Functional Enrichment Analysis of Differentially Expressed Genes Between Necroptosis-Related Patterns

After NMF clustering, to identify DEGs between two different necroptosis phenotypes, we used the “limma” package in R to evaluate gene expression differences through T statistics and *p* values (*p* < 0.001) calculated by empirical Bayesian estimation in the linear model (Ritchie et al., 2015). Then, we used Gene Ontology (GO) and Kyoto Encyclopedia of Genes and Genomes (KEGG) pathway enrichment analyses of DEGs between necroptosis-related patterns through the “clusterProfiler,” “enrichplot,” and “DOSE” R packages. The GO terms were in the biological process (BP), cellular component (CC), and molecular function categories (MF). The results were visualized with the “ggplot2” R package.

### Establishment and Validation of the NRG Prognostic Signature

Based on the prognosis-related NRGs in the univariate Cox regression model, the “glmnet” R package performed the least absolute shrinkage and selection operator (LASSO) and selected the minimum criteria to identify important prognostic genes, which contained 16 NRGs (Supplementary Table S2). Eventually, the multivariate Cox regression made the NRG signature more optimized and practical, with 10 NRGs remaining. In addition, the NRGscore formula was obtained as follows:

NRGscore =Σ(exp Genei×coefficient Genei ) .

After calculating the optimal cutoff of NRGscore by the “surv_cutpoint” function in R, we divided TCGA-KIRC cohort into high- and low-risk groups. With the help of Kaplan–Meier analysis (“survival” package) and receiver operating characteristic (ROC) curve (“timeROC” package), the predictive ability of the prognostic model was assessed. The ROC curve was quantified with the area under the curve (AUC). The same NRGscore calculation formula, cutoff value, and analysis methods were applied in the E-MTAB-1980 cohort to validate the signature.

### Establishment of the Nomogram Model

A nomogram is an intuitive clinical prognosis prediction model integrating a variety of prognosis-related variables. We established a nomogram model to provide a more accurate prediction of prognosis for clinical patients based on NRGscore and clinicopathological characteristics. First, univariate Cox regression analysis was utilized to evaluate predicted values of variables. Then, the coefficient was further determined *via* multivariate Cox regression analysis. The “rms” R package then established a nomogram for predicting the operating system. In addition, we used the “DynNom” R package to construct a dynamic nomogram to visualize the model. Concordance index (C-index) and calibration analysis were applied to estimate the accuracy and consistency. Finally, the clinical application value of the nomogram was evaluated using decision curve analysis (DCA).

### Evaluation of the Efficacy of Chemotherapy and Targeted Drugs

The chemotherapeutic response of KIRC patients was evaluated by Genomics of Drug Sensitivity in Cancer (GDSC) (https://www.cancerRxgene.org). Eight chemotherapeutic and targeted drugs in KIRC treatment were chosen, including axitinib, bortezomib, cisplatin, gefitinib, sorafenib, sunitinib, temsirolimus, and vinblastine. The ridge regression algorithm was used to calculate the half-maximal inhibitory concentration (IC_50_), and satisfactory prediction accuracy was obtained through 10-fold cross-validation (Geeleher et al., 2014). The process was calculated by the “pRRophetic” R package.

### Statistical Analysis

All statistical analyses were completed with R software (version 4.0.4) in this study. The Wilcoxon rank-sum test or paired-samples t-test was used to verify the statistical difference in two groups. When comparing the difference among more than two groups, the Kruskal–Wallis test was selected. Spearman’s correlation analysis calculated the correlation coefficients between TMB, immune checkpoint gene expression, and NRGscore. The “maftools” package was used to build waterfall plots to show the frequency of gene mutations. *P*-value <0.05 was set as a statistically significant standard.

## Result

**FIGURE 1:**
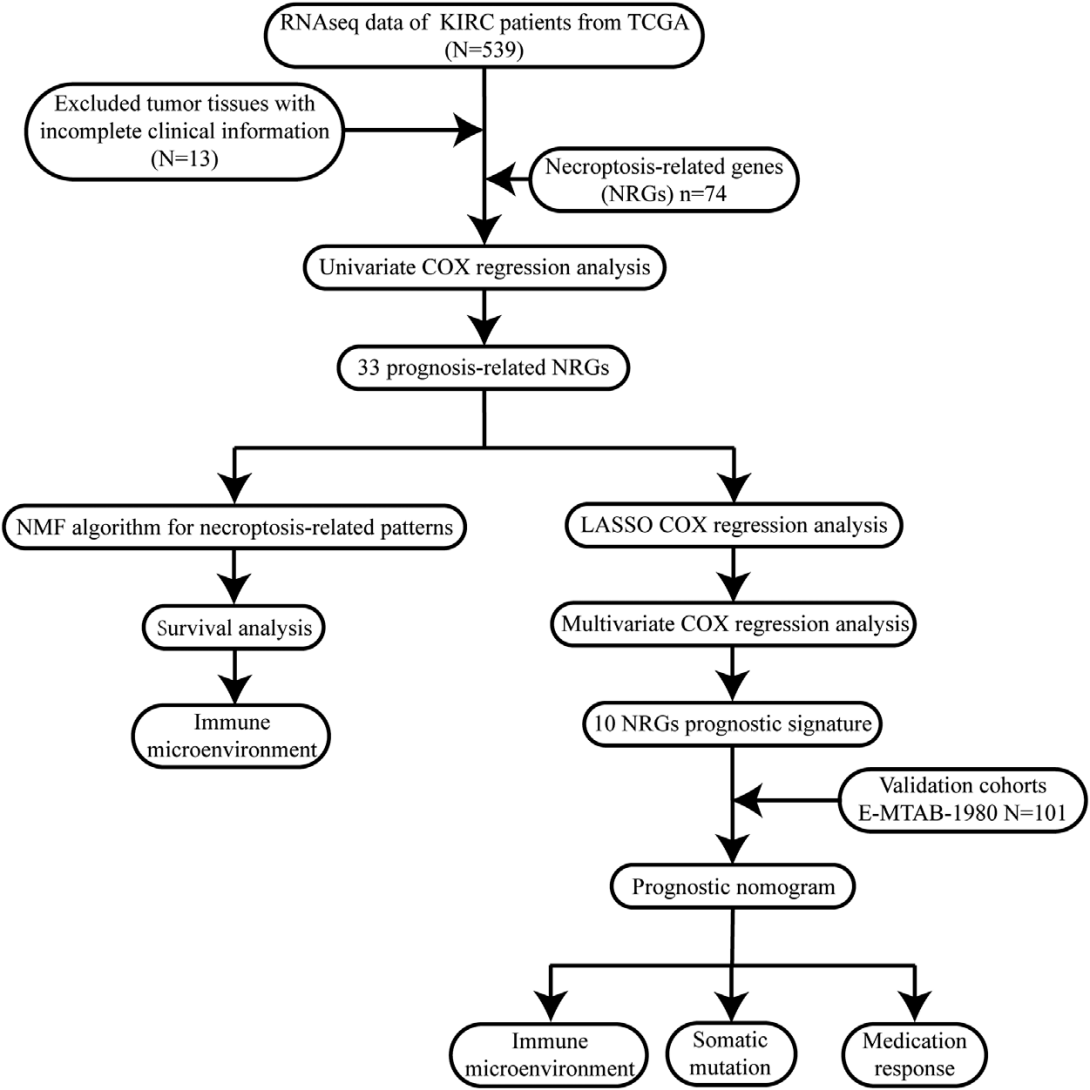
Flow chart of our study.

To describe our research intuitively and systematically, we show the research process in Figure 1.

### NMF Clustering of Necroptosis-Related Patterns in KIRC

**FIGURE 2:**
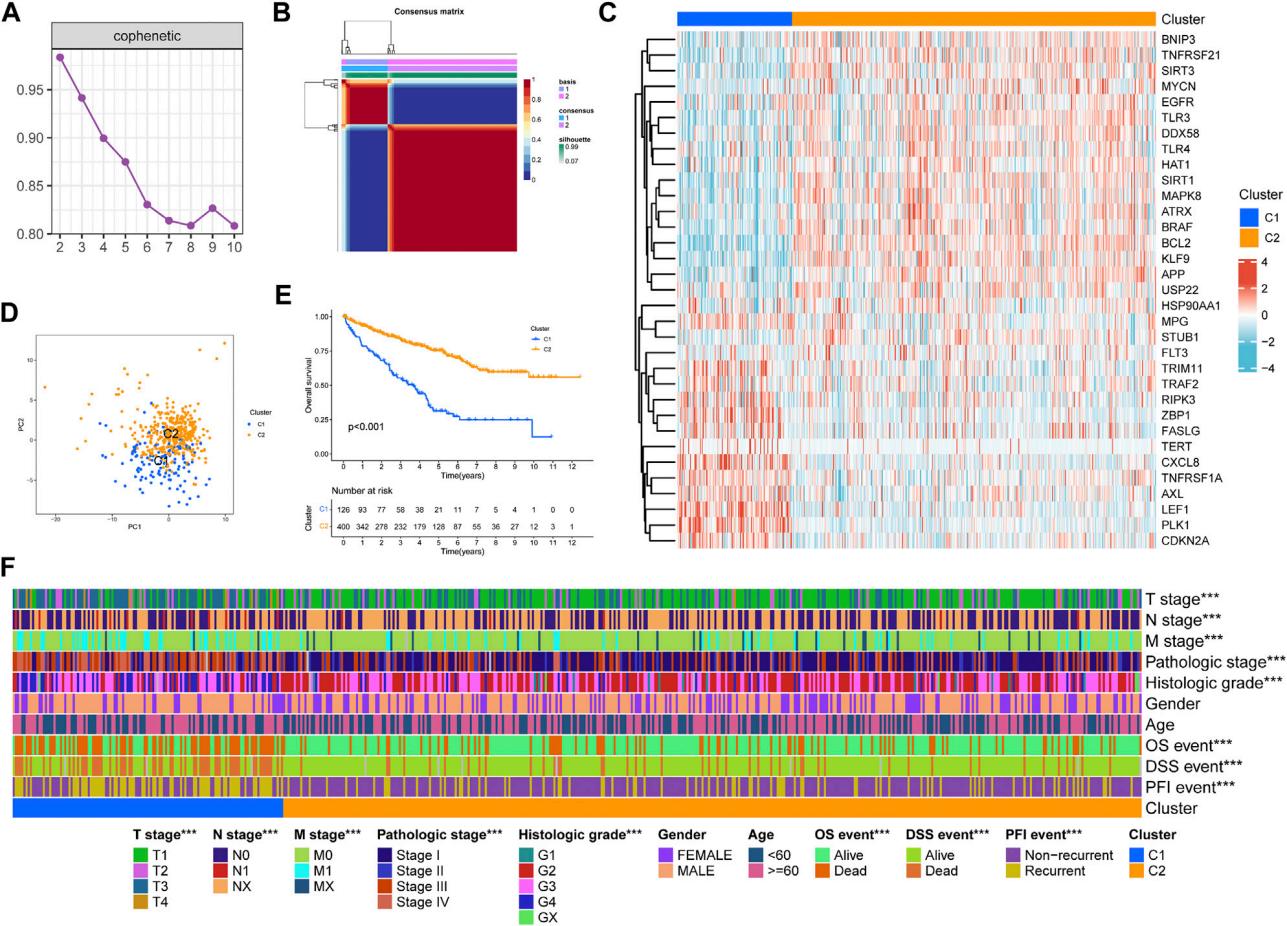
Non-negative matrix factorization clustering of necroptosis-related patterns in TCGA-KIRC cohort. **(A)** Cophenetic coefficients. **(B)** Consensus matrix heatmap when k = 2. **(C)** Expression profile of prognosis-related NRGs. PCA analysis **(D)** and Kaplan–Meier analysis **(E)** of necroptosis-related patterns. **(F)** Clinical relevance of necroptosis-related patterns. *p < 0.05; **p < 0.01; ***p < 0.001.

We performed NMF clustering on TCGA-KIRC cohort based on 33 prognosis-related NRGs in the univariate COX regression model (Table 1). According to cophenetic coefficients, k = 2 was the best clustering result (Figures 2A,B). Eventually, we identified two distinct necroptosis-related patterns termed necroptosis. C1 (*n* = 126) and necroptosis. C2 (*n* = 400). Figure 2C showed a transcription profile heatmap of 33 prognosis-related NRGs in C1 and C2. Afterward, we performed PCA to further complement the distinction between C1 and C2 at NRG transcription levels (Figure 2D). Kaplan–Meier analysis indicated that C2 had significantly longer OS than C1 (Figure 2E, *p* < 0.001). Ultimately, the chi-square test was used to reveal the distinction in clinicopathological characteristics between C1 and C2 (Figure 2F). As shown in the figure, the distribution of TNM stages, pathologic stage, histologic grade, OS, DSS, and PFI events was significantly distinct in C1 and C2. In addition, the advanced pathological characteristics and bad prognosis results had a tendency to concentrate on C1.

**TABLE 1:** Prognosis-related NRGs selected by univariate COX regression analysis.

Gene	HR	z	*p*-value
PLK1	1.979299	9.051351	1.41E-19
BCL2	0.621209	^−^6.31464	2.71E-10
KLF9	0.593631	^−^6.20142	5.60E-10
APP	0.568731	^−^5.56018	2.69E-08
ZBP1	1.579254	4.719934	2.36E-06
CDKN2A	1.562045	4.677732	2.90E-06
TNFRSF21	0.714626	−4.55388	5.27E-06
CXCL8	1.236663	4.489611	7.14E-06
MYCN	0.546308	^−^4.3527	1.34E-05
SIRT1	0.537114	^−^4.34934	1.37E-05
TLR3	0.744846	^−^4.30921	1.64E-05
TNFRSF1A	2.102905	3.896019	9.78E-05
MAPK8	0.459532	^−^3.75792	0.000171
MPG	1.778068	3.732143	0.00019
TRIM11	2.37506	3.701839	0.000214
BNIP3	0.734319	^−^3.62944	0.000284
TLR4	0.706861	^−^3.61795	0.000297
ATRX	0.542251	^−^3.53318	0.000411
BRAF	0.551977	^−^3.46673	0.000527
TRAF2	1.765383	3.347284	0.000816
LEF1	1.224713	3.28036	0.001037
DDX58	0.780107	^−^2.89537	0.003787
USP22	0.677291	^−^2.68993	0.007147
SIRT3	0.558047	^−^2.62101	0.008767
AXL	1.290534	2.593384	0.009504
HAT1	0.602383	^−^2.49452	0.012613
HSP90AA1	0.705533	^−^2.46741	0.013609
STUB1	0.720338	^−^2.31529	0.020597
FLT3	0.751125	^−^2.27881	0.022679
TERT	1.31171	2.251203	0.024373
FASLG	1.171632	2.107158	0.035104
EGFR	0.854597	^−^2.09633	0.036053
RIPK3	1.371085	1.980909	0.047601

HR, hazard ratio.

### Tumor Immune Microenvironment of Necroptosis-Related Patterns

**FIGURE 3:**
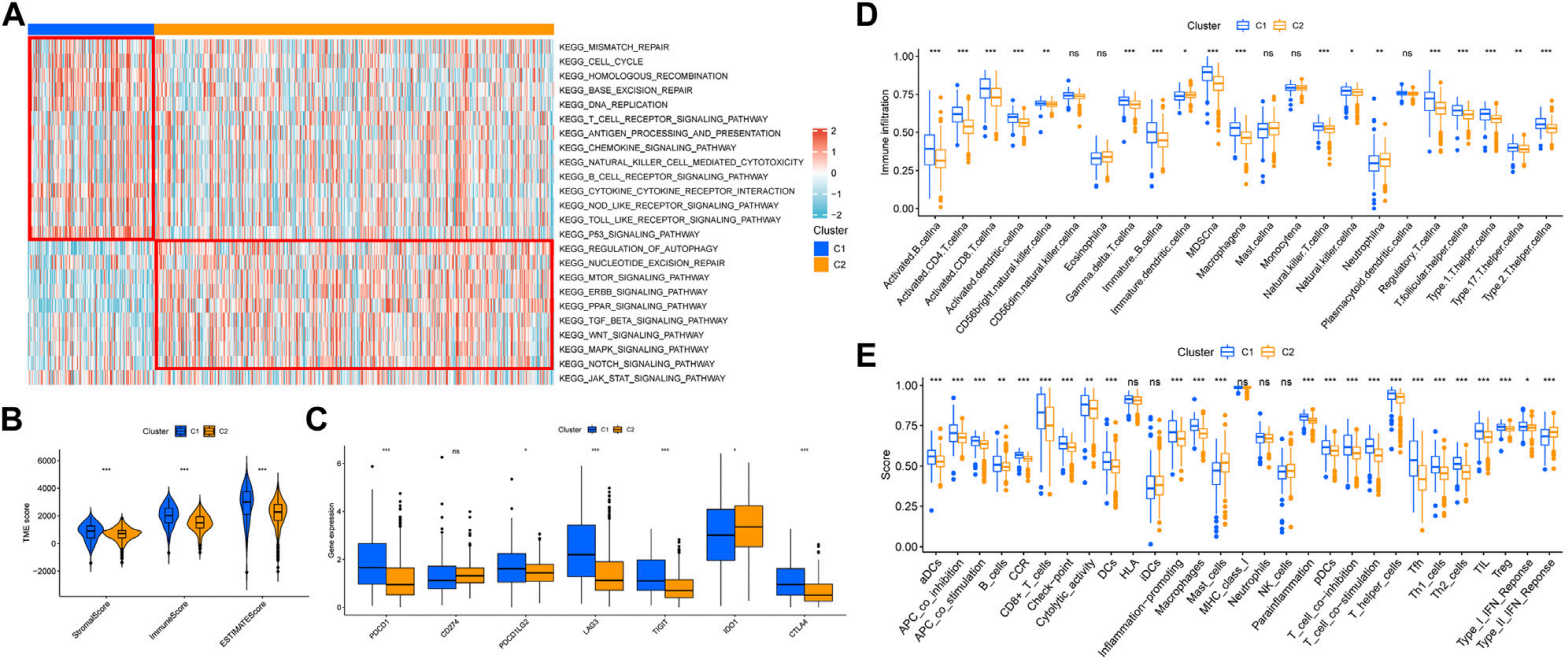
Correlation between necroptosis-related patterns and the tumor immune microenvironment. **(A)** Heatmap of GSEA analysis results. **(B)** Differential analysis of stromal, immune, and ESTIMATE scores. **(C)** Differential analysis of the expression of immune checkpoints. **(D)** Infiltration of 23 TIICs in necroptosis-related patterns. **(E)** Enrichment scores of immune-related functions in necroptosis-related patterns. *p < 0.05; **p < 0.01; ***p < 0.001; ns = no significance.

Through GSEA analysis, we confirmed the concentration level of TCGA-KIRC samples in DNA damage repair, immune activation, stromal score, and carcinogenic-related pathways (Figure 3A). We believed that C1 had a higher expression in DNA damage repair and immune activation–related pathways, while C2 had significantly higher concentration in carcinogenic-related pathways, including regulation of autophagy. According to previous studies, the process of necroptosis shows a strong inflammatory response. To distinguish the difference between C1 and C2 in immune-related characteristics, we first quantified the tumor microenvironment composition using ESTIMATE (Figure 3B). The stromal score (*p* < 0.001), immune score (*p* < 0.001), and ESTIMATE score (*p* < 0.001) of C1 were all significantly higher than those in C2. Then, we compared the distinctions between TIICs and immune-related functions between necroptosis-related patterns through ssGSEA. Moreover, the expression of multiple immune checkpoints, including PDCD1, PDCD1LG2, LAG3, TIGIT, and CTLA4, was significantly higher in C1 (Figure 3C). ssGSEA analysis revealed that B cells, T cells, DCs, macrophages, and neutrophils in C1 infiltrated significantly higher than those in C2 (Figure 3D). Consistently, almost all immune functions in C1 were expressed higher (Figure 3E).

**FIGURE 4:**
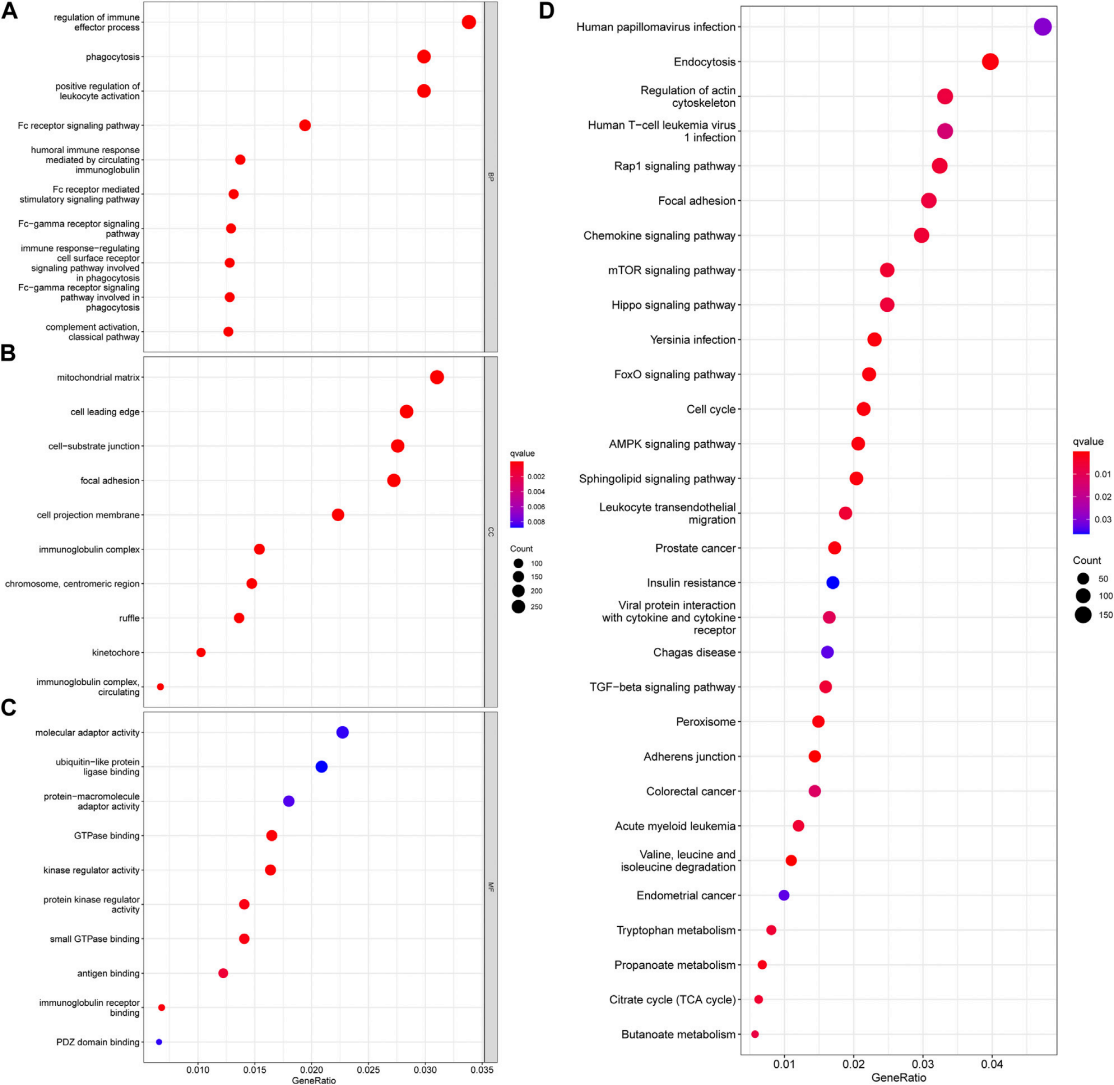
Functional enrichment analyses of DEGs between necroptosis-related patterns. **(A)** Biological process, **(B)** cellular component, **(C)** molecular function, and **(D)** KEGG pathways.

In addition, functional enrichment analyses of DEGs between necroptosis-related patterns were applied to explore differences at the molecule. GO analysis indicated that DEGs were mainly involved in the regulation of the immune effector process, phagocytosis, positive regulation of leukocyte activation, and multiple immune-related biological processes (Figure 4A). Transcription proteins were mostly located in mitochondrial matrix, cell leading edge, and cell−substrate junction (Figure 4B). Molecular functions were mostly concentrated in molecular adapter activity, ubiquitin−like protein ligase binding, and protein−macromolecule adapter activity (Figure 4C). In addition, the DEGs were related to several immune-related pathways, such as the chemokine signaling pathway, mTOR signaling pathway, and TGF-β signaling pathway (Figure 4D).

### Establishment of the NRG Signature in TCGA-KIRC Cohort

**FIGURE 5:**
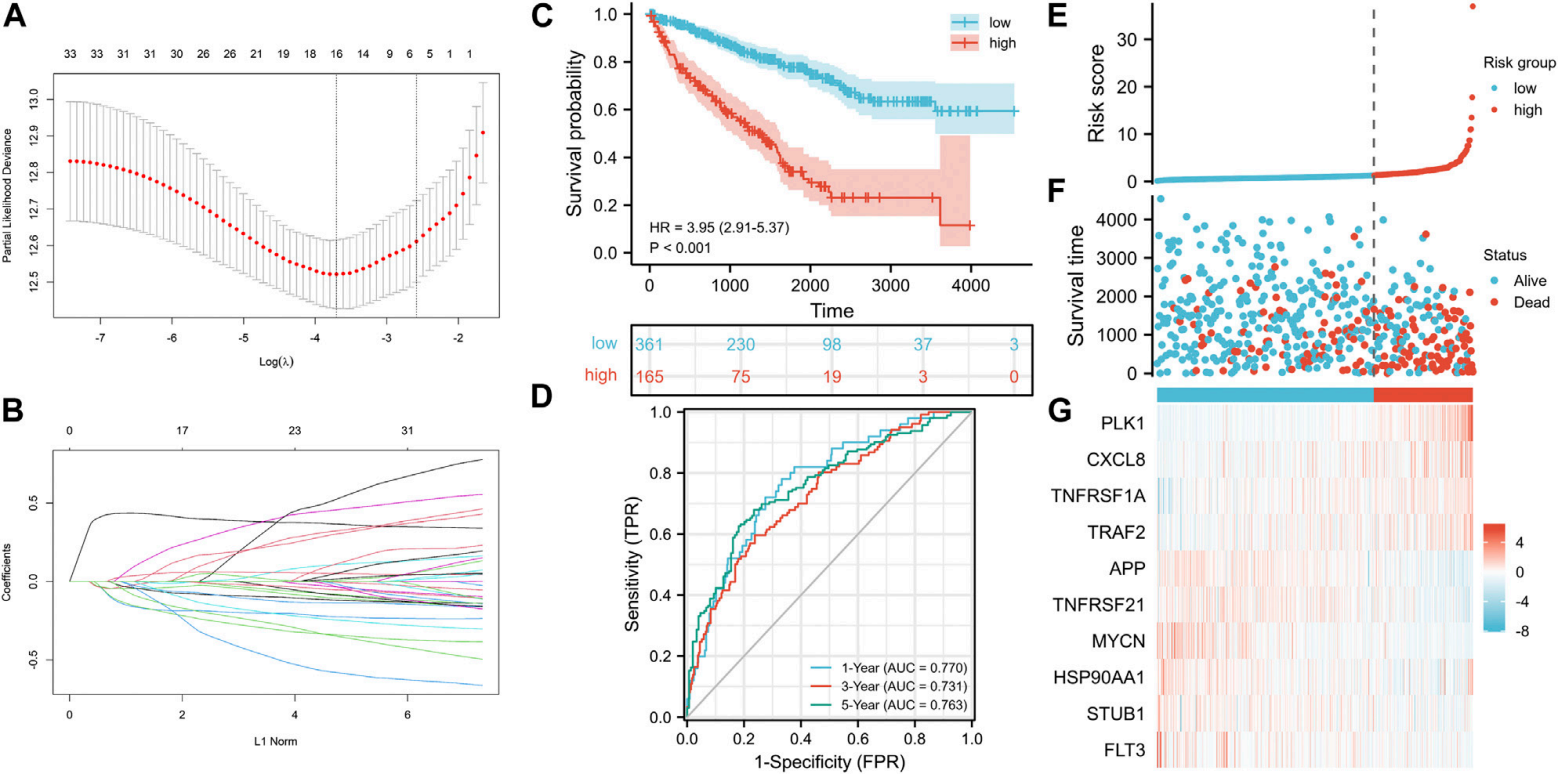
Establishment of the NRG signature based on the training set. **(A, B)** LASSO COX regression analysis. **(C)** Kaplan–Meier analysis between NRGscore-defined groups. **(D)** Time-dependent ROC curve of NRGscore. **(E)** NRGscore distribution. **(F)** Survival status heatmap. **(G)** NRG expression profile heatmap.

We established a NRG prognostic signature to obtain an indicator that could accurately and effectively predict the clinical survival rate of KIRC patients. In previous studies, we have obtained a univariate COX regression analysis model with 33 NRGs. Then, the univariate Cox regression model was processed to obtain the coefficient through LASSO Cox regression analysis, and the minimum standard was selected to further screen 16 genes (Figures 5A,B). The model was eventually optimized using multivariate Cox regression analysis, with a total of 10 genes remaining, including PLK1, APP, TNFRSF21, CXCL8, MYCN, TNFRSF1A, TRAF2, HSP90AA1, STUB1, and FLT3. We also obtained a quantitative indicator: NRGscore = (0.39839 × PLK1 expression)—(0.21626 × APP expression)—(0.13856 × TNFRSF21 expression) + (0.08438 × CXCL8 expression)—(0.31476 × MYCN expression) + (0.40884 × TNFRSF1A expression) + (0.39387 × TRAF2 expression)—(0.26223 × HSP90AA1 expression)—(0.48853 × STUB1 expression)—(0.25716 × FLT3 expression). Then, we calculated NRGscore for each patient based on the abovementioned formula. In Kaplan–Meier analysis, we divided patients into the high-risk group (n = 165) and low-risk group (n = 361) based on the optimal cutoff value (cut point = 1.276099) for NRGscore. In addition, the result revealed that the OS of patients in the high-risk group was significantly worse than that in the low-risk group [Figure 5C, hazard ratio (HR) = 3.95 (2.91–5.37), *p* < 0.001]. Additionally, we used ROC curves to assess the veracity of NRGscore to predict the OS survival rate of KIRC patients. The AUCs for the 1-, 3-, and 5-year OS survival rates were 0.770, 0.731, and 0.763, respectively (Figure 5D). Figures 5E–G showed that the proportion of deaths in the high-risk group was elevated and increased with NRGscore. The expression of PLK1, CXCL8, TNFRSF1A, and TRAF2 was increased with the risk processes, whereas APP, TNFRSF21, MYCN, HSP90AA1, STUB1, and FLT3 were negatively correlated with NRGscore.

### Validation of the NRG Signature in the E-MTAB-1980 Cohort

**FIGURE 6:**
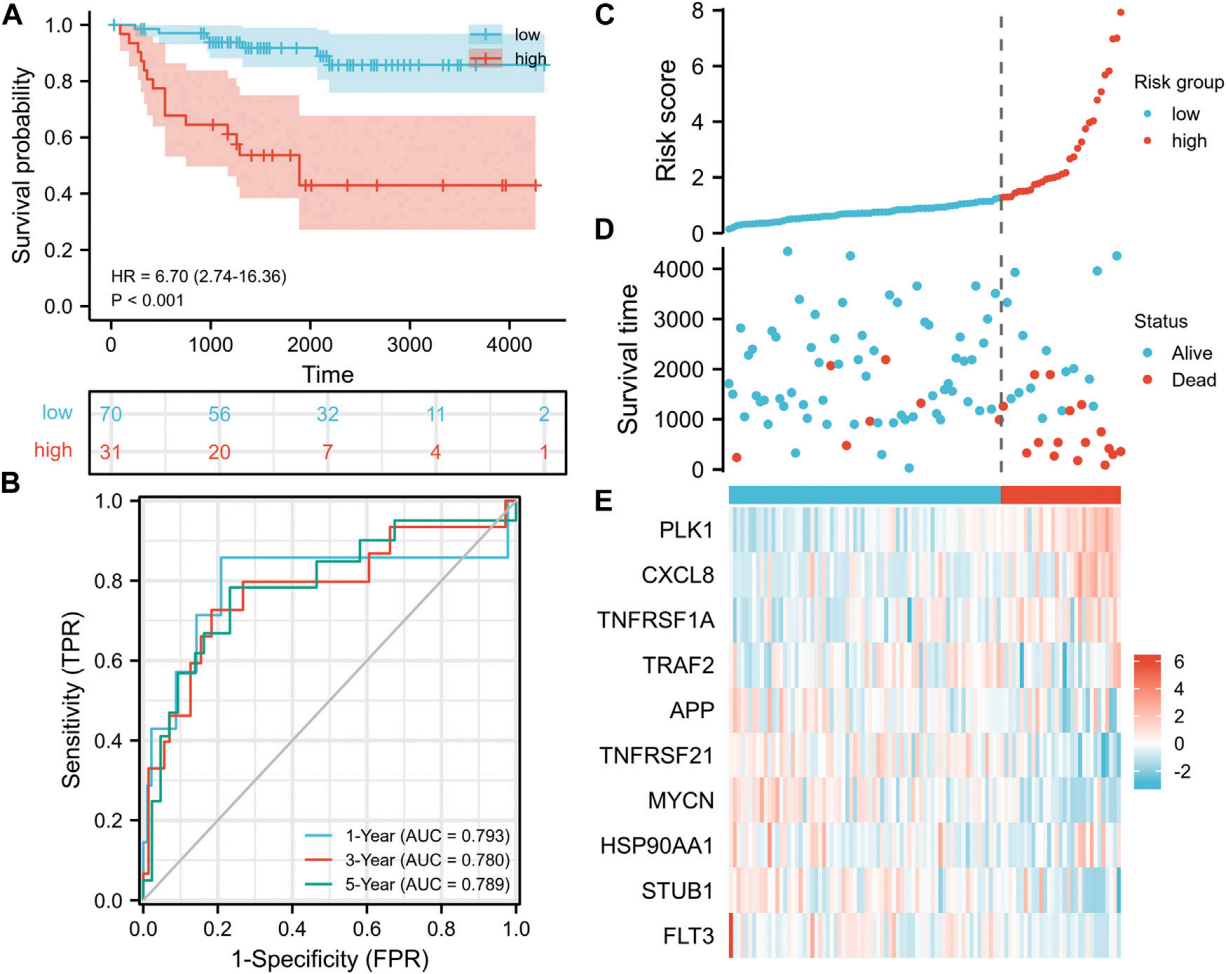
Validation of the NRG signature based on the test set. **(A)** Kaplan–Meier analysis between NRGscore-defined groups. **(B)** Time-dependent ROC curve of NRGscore. **(C)** NRGscore distribution. **(D)** Survival status heatmap. **(E)** NRG expression profile heatmap.

To further verify the stability and accuracy of NRGscore in KIRC patients, we used 101 KIRC patients in E-MTAB-1980 as the test set. We quantified samples in the test set using the same NRGscore calculation formula and grouped them with the same cutoff value (cut point = 1.276099) as the training set [high-risk group (*n* = 31) and low-risk group (*n* = 70)]. Kaplan–Meier analysis showed that a high NRGscore indicated significantly poor OS [Figure 6A, hazard ratio (HR) = 6.70 (2.74–16.36), *p* < 0.001]. ROC curves showed favorable results that the AUCs were 0.793 at a 1-year OS survival rate, 0.780 at a 3-year OS survival rate, and 0.789 at a 5-year OS survival rate (Figure 6B). The risk score distribution, survival status, and expression profile heatmaps showed a trend similar to that of the training set (Figures 6C–E).

To illustrate the superiority of the NRG signature, we compared the other two immune-autophagy–related gene signature (Zhang et al., 2021) and pyroptosis-related gene signature (Sun et al., 2021) recently published. After obtaining the genes constituting the prognosis signature from literature, the Kaplan–Meier curves and ROC curves were constructed by TCGA-KIRC cohort (Supplementary Figure S1). According to the results, the NRG signature had better prediction accuracy for the OS of KIRC patients.

### Clinical Relevance of the NRG Signature

**FIGURE 7:**
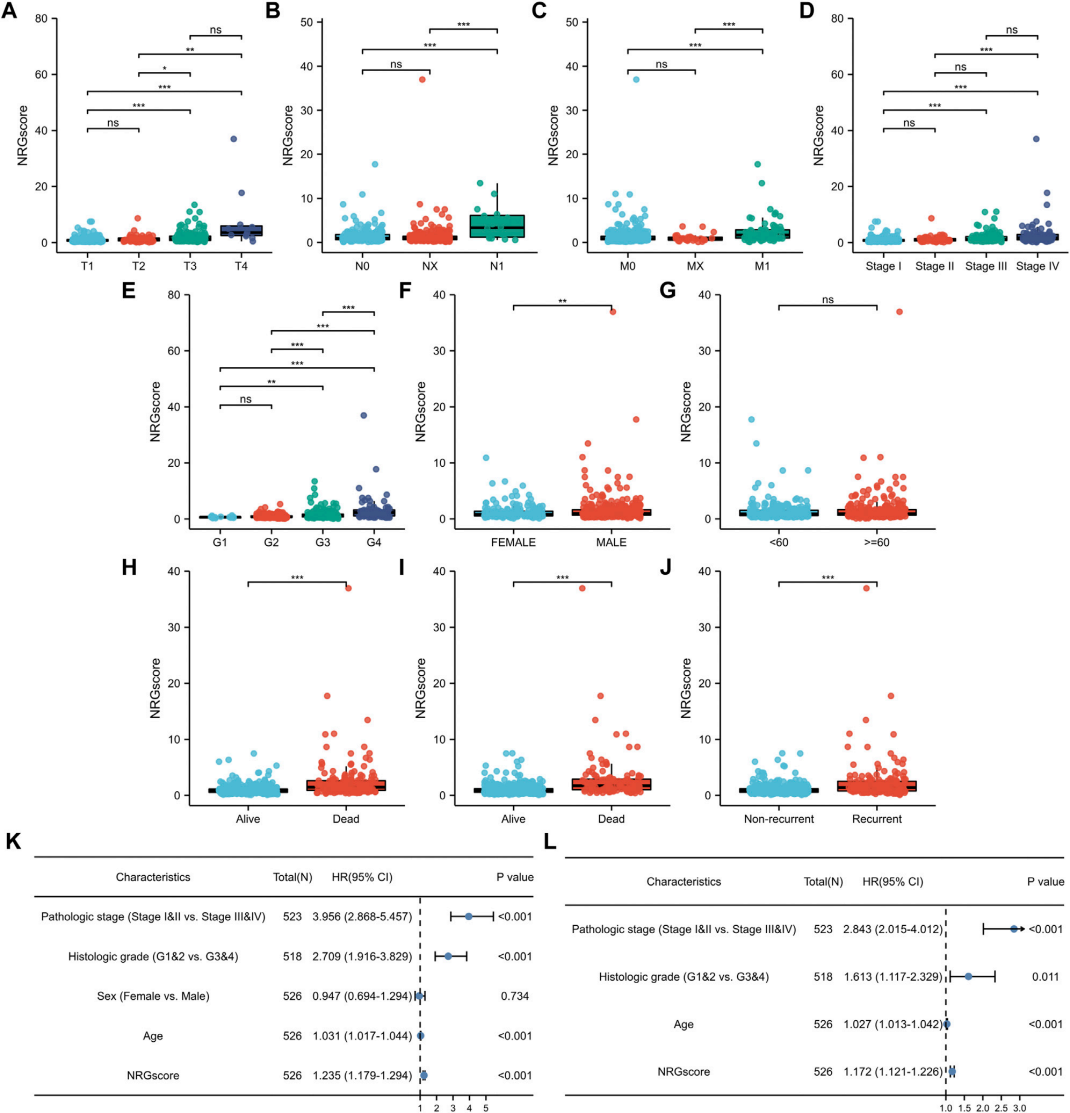
Clinical relevance of the NRG signature. **(A–J)** NRGscore differences between subgroups of clinicopathological parameters, including T stage **(A)**, N stage **(B)**, M stage **(C)**, pathologic stage **(D)**, histologic grade **(E)**, gender **(F)**, age **(G)**, OS event **(H)**, DSS event **(I),** and PFI event **(J)**. Univariate **(K)** and multivariate **(L)** Cox regression analysis of NRGscore and clinicopathological parameters. *p < 0.05; **p < 0.01; ***p < 0.001; ns = no significance.

We calculated the correlation between NRGscore and clinicopathological characteristics for further analysis of the clinical benefits of the NRG signature. It can be seen that NRGscore increased significantly with the progress of TNM stages, pathologic stage, and histologic grade (Figures 7A–E). Male patients also scored higher than female patients (Figure 7F). There was no statistical difference between the age group (Figure 7G). In addition, the high NRGscore indicated a higher incidence of bad OS, DSS, and PFI events (Figures 7H–J).

Next, we applied univariate and multivariate Cox regression analyses to investigate whether NRGscore was an independent prognostic indicator of KIRC patients. Univariate Cox regression analysis pointed out that pathologic stage, histologic grade, age, and NRGscore were hazard factors (Figure 7K). Then, multivariate Cox regression analysis verified that NRGscore could be utilized as a robust independent prognostic indicator for KIRC patients [hazard ratio (HR) = 1.180 (1.127–1.235), *p* < 0.001, Figure 7L].

### Construction of a Nomogram Model Based on the NRG Signature

**FIGURE 8:**
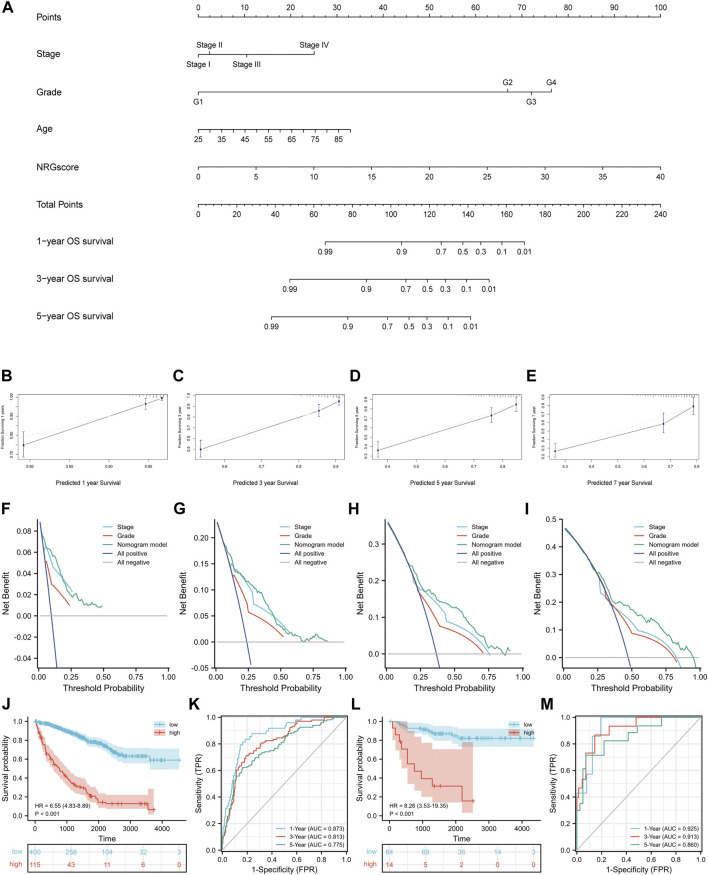
Construction of the nomogram model. **(A)** Nomogram for predicting the OS probability over 1, 3, and 5 years **(B–E)** Calibration curves for evaluating the fitness of the nomogram model in 1, 3, 5, and 7 years. **(F–I)** DCA curves of 1, 3, 5, and 7 years. Kaplan–Meier analysis **(J)** and time-dependent ROC curves **(K)** of the nomogram model in TCGA-KIRC cohort. Validation of the nomogram model in the E-MTAB-1980 cohort with the Kaplan–Meier analysis **(L)** and time-dependent ROC curves **(M)**.

Next, according to the results of Cox regression analyses, we integrated NRGscore with several clinicopathological characteristics, including pathologic stage, histologic grade, and age, to construct a nomogram model that can more accurately and steadily evaluate the OS survival probability of patients in TCGA-KIRC cohort (Figure 8A). A total of 515 KIRC patients with complete clinicopathologic information were included in the model analysis. Then, C-index and calibration curves were utilized to assess the precision of the nomogram model. The C-index reached 0.771 (95% CI: 0.736–0.807, *p* < 0.0001). The calibration curves also confirmed that the nomogram model possessed excellent accuracy (Figures 8B–E). In addition, we used DCA curves to prove that NRGscore has a better clinical application value for patient OS prediction than the pathologic stage and histologic grade (Figures 8F–I). Finally, we defined patients in TCGA-KIRC cohort as high or low risk according to the optimal cutoff nomogram score (cut point = 0.7666527). Kaplan–Meier analysis suggested that high-risk patients showed poorer OS than low-risk patients [Figure 8J, hazard ratio (HR) = 6.55 (4.83–8.89), *p* < 0.001]. AUCs were 0.873 at the 1-year OS survival rate, 0.813 at the 3-year OS survival rate, and 0.775 at the 5-year OS survival rate (Figure 8K). We also validated the nomogram model using the E-MTAB-1980 cohort. The test set used the same model and cutoff value. Kaplan–Meier analysis showed the same result as the training set [Figure 8L, hazard ratio (HR) = 8.26 (3.53–19.35), *p* < 0.001]. In addition, the 1-, 3-, and 5-year AUCs were 0.925, 0.913, and 0.860, respectively (Figure 8M).

### Coexpression Relevance and GSEA

**FIGURE 9:**
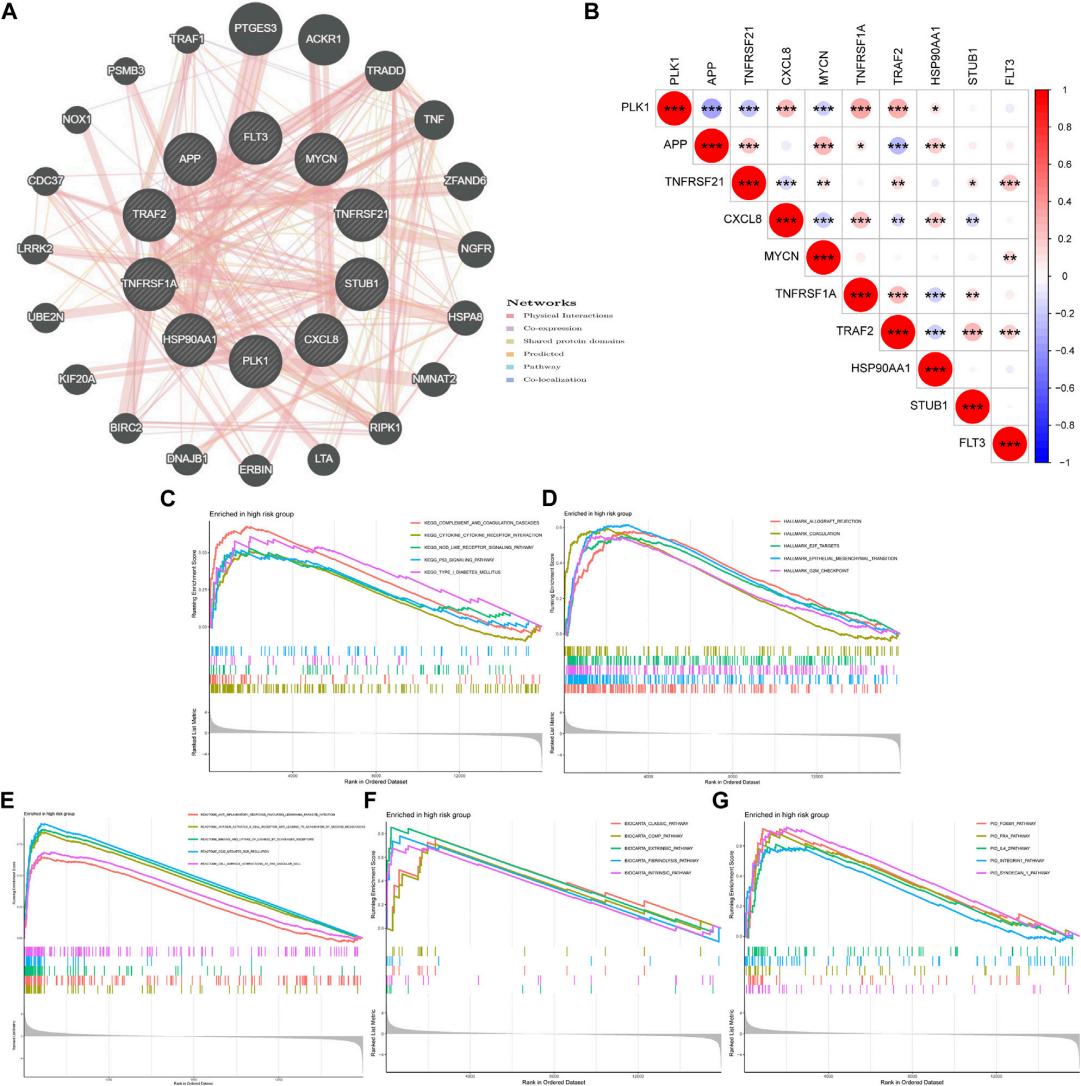
Coexpression relevance and GSEA of the NRG signature. **(A)** Regulatory network of 10 signature-related NRGs and conceivable interaction proteins built by GeneMANIA. **(B)** Coexpressed correlation of 10 model-related NRGs in KIRC. **(C–G)** GSEA analyses based on KEGG **(C)**, Hallmark **(D)**, Reactome **(E)**, BioCarta **(F)**, and PID **(G)** gene sets in the high-risk group. *p < 0.05; **p < 0.01; ***p < 0.001.

We used GeneMANIA to predict and visualize the interaction networks of the 10 NRGs that comprise NRGscore and potential interactive molecules (Figure 9A) (Warde-Farley et al., 2010). GeneMANIA automatically identifies genes that contained several hub genes for necroptosis, including RIPK1, TNF, BIRC2, and CDC37. Figure 9B showed the coexpressed correlation of 10 NRGs in KIRC. TRAF2 had the highest number of NRGs with significant coexpression correlation (*n* = 8).

Then, GSEA was used to explore potential biological processes and signal pathways in the high-risk group of TCGA-KIRC cohort. GSEA based on the KEGG gene set suggested that carcinogenic and immune-related pathways were highly concentrated, including complement and coagulation cascades, cytokine–cytokine receptor interaction, NOD-like receptor (NLR) signaling pathway, and P53 signaling pathway (Figure 9C). The NLR signaling pathway plays a regulatory role in inflammation-related cancer and can be used as a therapeutic target (Liu et al., 2019). The transcription factor p53 is an important tumor suppressor. A p53 activating compound has been proven to be significantly cytotoxic to breast cancer and colon cancer cells (Mirgayazova et al., 2019). In addition, Hallmark gene sets of cell cycles and epithelial–mesenchymal transition were also highly expressed (Figure 9D). In addition, Figures 9E–G indicated that the high-risk group was related to immune-related reactions, classic pathways, and coagulation pathways.

### Correlation Between the NRG Signature and Tumor Immune Microenvironment

**FIGURE 10:**
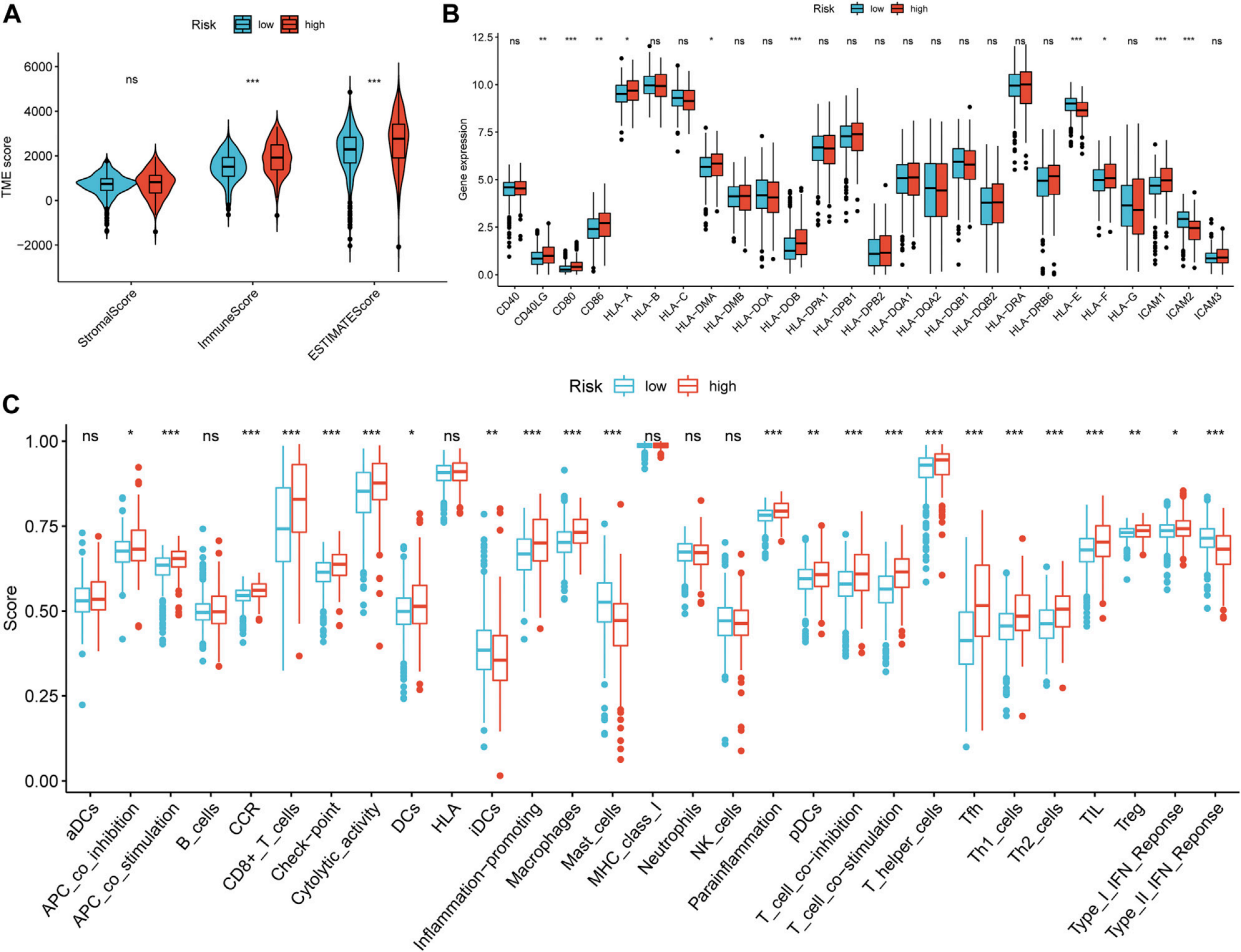
Correlation between the NRG signature and tumor immune microenvironment. **(A)** Differential analysis of stromal, immune, and ESTIMATE scores between NRGscore-defined groups. **(B)** Differential analysis in the expression of MHC molecules, costimulatory molecules, and adhesion molecules between NRGscore-defined groups. **(C)** Enrichment scores of immune-related functions in NRGscore-defined groups. *p < 0.05; **p < 0.01; ***p < 0.001; ns = no significance.

As a result of the strong inflammatory response of necroptosis reported in previous studies and the distinction in immunophenotype between necroptosis-related patterns, we further analyzed the correlation between the NRG signature and TIME. First, we evaluated the distinction in TME scores between NRG-defined groups with the ESTIMATE algorithm (Figure 10A). The Wilcoxon rank-sum test suggested that immune score (*p* < 0.001) and ESTIMATE score (*p* < 0.001) in the high-risk group were significantly higher than those in the low-risk group. Figure 10B indicated that the expression of costimulatory molecules, except CD40, was significantly elevated in the high-risk group. As for adhesion molecules, ICAM1 and ICAM2 were highly expressed in high- and low-risk groups, respectively. Moreover, the expression levels of most major histocompatibility complex (MHC) molecules had no statistical difference in NRGscore-defined groups. ssGSEA showed that most immune-related functions were highly concentrated in the high-risk group (Figure 10C). Consistently, there was no significant distinction in antigen presentation between NRGscore-defined groups.

**FIGURE 11:**
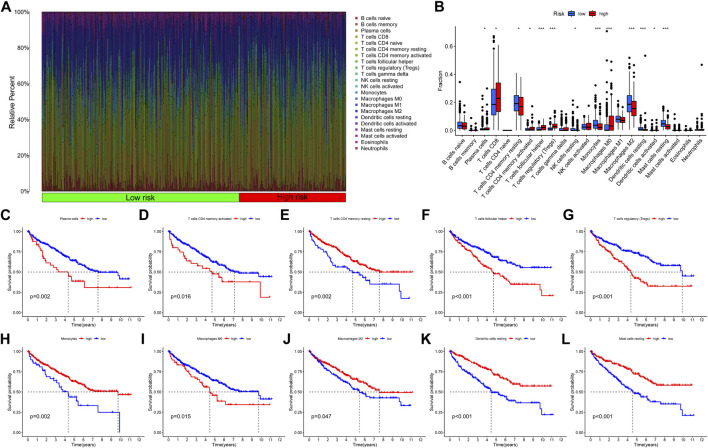
Correlation between the fraction of 22 TIICs and the NRG signature on the basis of the CIBERSORT algorithm. **(A)** Proportion of 22 TIICs in KIRC. **(B)** Differential analysis of 22 TIIC fractions between NRGscore-defined groups. **(C–L)** Association between the infiltration level of TIICs **[**plasma cells **(C)**, activated CD4 memory T cells **(D)**, resting CD4 memory T cells **(E)**, follicular helper T cells **(F)**, Tregs **(G)**, monocytes **(H)**, M0 macrophages **(I)**, M2 macrophages **(J)**, resting DCs **(K),** and resting mast cells **(L)**] and OS of KIRC patients. *p < 0.05; **p < 0.01; ***p < 0.001.

Then, we calculated the fraction of 22 TIICs in each TCGA-KIRC sample on the basis of the CIBERSORT algorithm. The results of a total of 415 samples were statistically significant. Figure 11A showed the distribution of TIICs in KIRC in the form of a grouping histogram. T cells and macrophages could be seen to account for the largest components. Next, we found that the fractions of plasma cells, CD8 T cells, activated CD4 memory T cells, follicular helper T cells, regulatory T cells (Tregs), M0 macrophages, and activated DCs were significantly higher in the high-risk group (Figure 11B), while resting CD4 memory T cells, resting natural killer (NK) cells, monocytes, M2 macrophages, resting DCs, and resting mast cells had lower fractions in the high-risk group (Figure 11B). Among these differentially distributed TIICs, higher fractions of plasma cells, activated CD4 memory T cells, follicular helper T cells, Tregs, and M0 macrophages and lower fractions of resting CD4 memory T cells, monocytes, M2 macrophages, resting DCs, and resting mast cells were significantly associated with poor OS survival probability in KIRC patients (Figures 11C−L). The abovementioned results suggested that necroptosis might affect the prognosis of KIRC patients through potential regulation of these TIICs.

### Correlation Between the NRG Signature and Somatic Mutation

**FIGURE 12:**
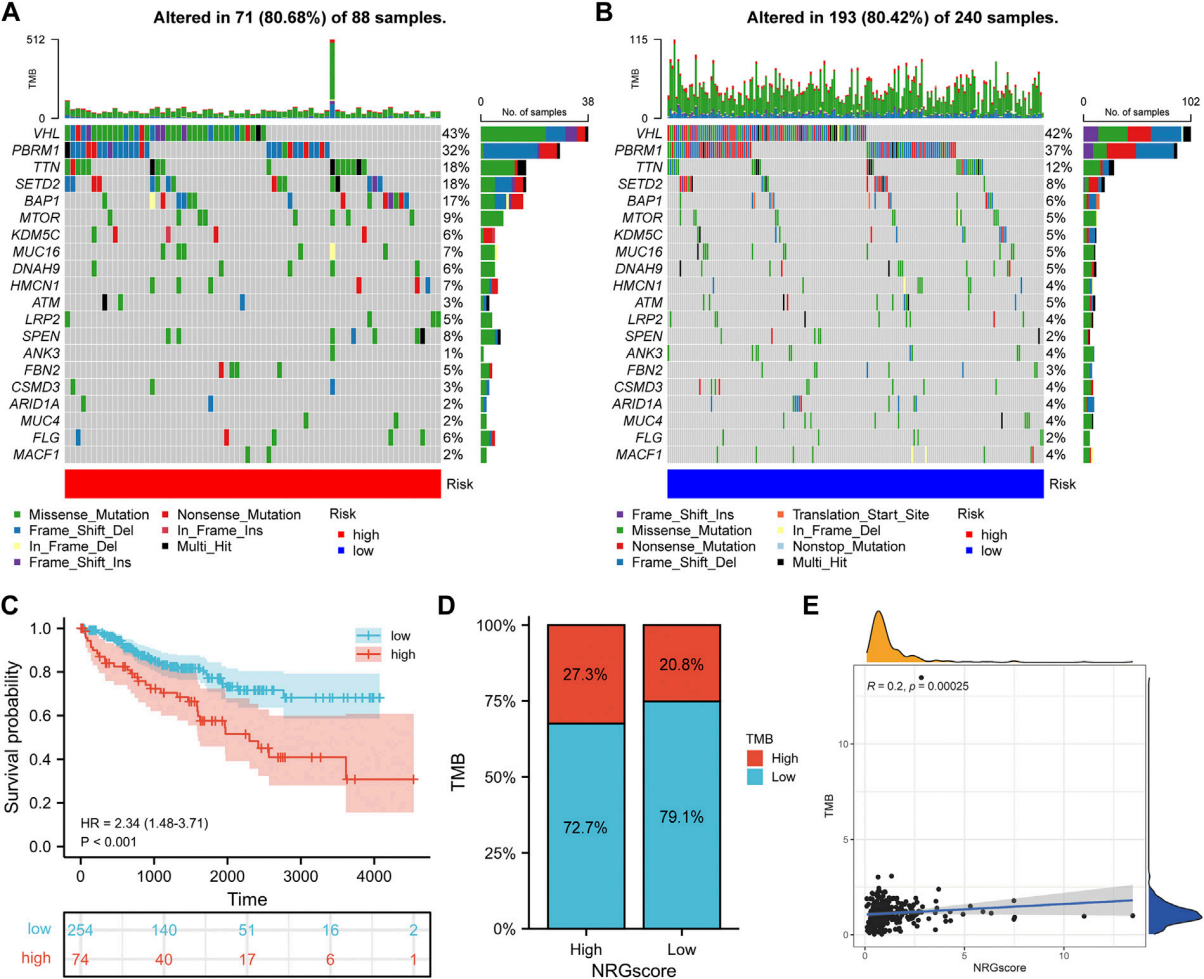
Correlation between the NRG signature and somatic mutation. **(A, B)** Waterfall plots of 20 genes with the highest mutation rate in the high-risk group **(A)** and low-risk group **(B)**. **(C)** Kaplan–Meier analysis of TMB in KIRC patients. **(D)** Distribution of the TMB level in NRGscore-defined groups **(E)** Correlation between NRGscore and TMB.

Tumorigenesis frequently occurs after accumulation of gene mutations (Martincorena and Campbell, 2015). Hence, we explored the distinction in somatic mutations between NRGscore-risk groups. The mutation spectrum and TMB of each sample in TCGA-KIRC were calculated on the basis of the single-nucleotide variation information. Waterfall plots showed that the 20 genes with the highest mutation rate in KIRC were VHL, PBRM1, TTN, SETD2, BAP1, MTOR, KDM5C, MUC16, DNAH9, HMCN1, ATM, LRP2, SPEN, ANK3, FBN2, CSMD2, ARID1A, MUC4, FLG, and MACF1 (Figures 12A,B). We applied the optimal TMB cutoff value to divide patients into low- and high-TMB groups. KIRC patients with higher TMB were associated with poorer OS survival probability (Figure 12C). As shown in Figure 12D, the proportion of high-TMB patients was higher in the high-risk group. In addition, we revealed a significant positive relevance between NRGscore and TMB in KIRC patients (Figure 12E, R = 0.2, *p* = 0.00025).

### Correlation Between the NRG Signature and Drug Sensitivity

**FIGURE 13:**
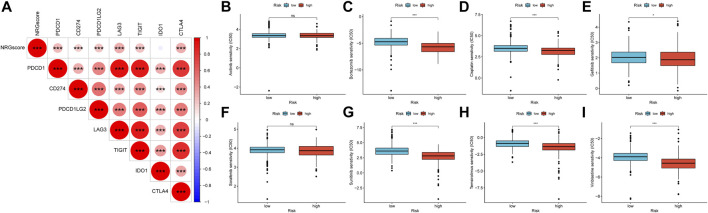
Therapeutic benefit of the NRGscore **(A)** Correlation between NRGscore and gene expression of seven immune checkpoints. **(B–I)** Correlation between the NRG signature and IC_50_ values of chemotherapy and targeted drugs, including axitinib **(B)**, bortezomib **(C)**, cisplatin **(D)**, gefitinib **(E)**, sorafenib **(F)**, sunitinib **(G)**, temsirolimus **(H)**, and vinblastine (**I**). *p < 0.05; **p < 0.01; ***p < 0.001; ns = no significance.

Recently, ICIs have gradually shown clinical benefits for advanced KIRC. However, because most patients showed no response to immunotherapy, it was important to find effective predictive markers. We calculated the correlation between NRGscore and gene expression of several immune checkpoints (Figure 13A). It was found that NRGscore was significantly positively correlated with the expression of PDCD1, CD274, PDCD1LG2, LAG3, TIGIT, and CTLA4, which indicated that patients in the high-risk group were more likely to benefit from immunotherapy.

The responsive predictive values of NRGscore for chemotherapy and targeted drugs were also calculated by IC_50_ values (Figures 13B–I). Compared with the low-risk group, the IC_50_ value of bortezomib, cisplatin, gefitinib, sunitinib, temsirolimus, and vinblastine was significantly lower in the high-risk group, which means patients with higher NRGscore were more sensitive to these drugs.

## Discussion

Necrosis was originally thought to be an uncontrolled form of accidental cell death, but a growing body of research has confirmed that necrosis can be induced and carried out in the form of apoptosis (Christofferson and Yuan, 2010; Linkermann and Green, 2014). This form of programmed cell death was called necroptosis. These activation factors include TNF-receptor superfamily, Toll-like-receptor superfamily, and interferon receptor (Khoury et al., 2020). But, unlike apoptosis cells, which maintain cell membrane integrity, necrosis-experiencing cells show damage to the cell membrane, leading to the release of immunogenic DAMPs, which in turn shows extreme immunogenicity (Häcker, 2000; Rosenbaum et al., 2009; Kaczmarek et al., 2013; Svensson et al., 2017). DAMPs can mediate the interaction between cancer cells and immune cells to trigger an anticancer-related immune response, such as the activation of cytotoxic CD8^+^ T lymphocytes, prompting DC to release proinflammatory cytokines and reduce Treg tumor immersion (Biswas and Mantovani, 2010; Werthmöller et al., 2015; Yatim et al., 2015; Sprooten et al., 2020). However, the immune landscape caused by necroptosis rarely is a one-way antitumor effect. For instance, IL-1α produced by necrotic tumor cells can directly stimulate the proliferation of neighboring cells and promote tumor progression (Grivennikov et al., 2010). The release of active nitrogen intermediates (RNI) and/or ROS associated with necrosis apoptosis may facilitate tumor development (Grivennikov et al., 2010). Therefore, further experimental research is needed to balance this complex immune landscape, through the necroptosis inducer in the *in vivo* tumor environment to achieve a “pure” protective effect, to achieve the purpose of precision immunotherapy. In addition, the detailed effects of necroptosis on KIRC are yet to be fully studied.

In this study, we identified two necroptosis-related patterns by NMF algorithm clustering. Necroptosis C1 showed a significantly poor OS survival probability. The proportion of patients in the advanced clinicopathological stages in high-risk necroptosis C1 was also significantly elevated. Furthermore, these two necroptosis-related patterns showed distinct biological pathway enrichment and TME immune cell infiltration. In TCGA-KIRC cohort, C1 was characterized by high levels of adaptive immunity activation and TME immune cell immersion. In addition, we found that several immune checkpoints (PD-1, PD-L2, LAG3, TIGIT, and CTLA4) were highly expressed in C1. Properly located and migrated T cells are the basis of tumor immune monitoring, but there was no matching survival advantage in C2. We speculated that the immunosuppressive microenvironment induced by high-level expression of immune checkpoint genes eliminated the antitumor effect based on activating the immune pathway and high infiltration level T cells (Dunn et al., 2002). The abovementioned evidence proved that necroptosis was of great significance in regulating the immune landscape of KIRC.

Then, we established a prognostic signature for predicting OS including 10 NRGs (PLK1, APP, TNFRSF21, CXCL8, MYCN, TNFRSF1A, TRAF2, HSP90AA1, STUB1, and FLT3) in TCGA-KIRC cohort to evaluate and quantify the necroptosis pattern of KIRC individuals (NRGscore). A series of analyses were carried out by NRGscore-defined groups. Survival analysis suggested that the OS of patients in the high-risk group should be significantly reduced. It was consistently and significantly confirmed in a separate external E-MTAB-1980 cohort. High NRGscore also indicated tumor progression or poor prognosis event. Univariate and multivariate Cox analyses proved that NRGscore could be utilized as an independent prognostic marker. Among the 10 NRGs included in the prognostic signature, PLK1 could promote proliferation and inhibit apoptosis in KIRC cells, and had been proven to be upregulated and inhibit necroptosis in hormone-resistant prostate cancer (Deeraksa et al., 2013; Gao et al., 2020). The compound of APP and death receptor 6 (DR6/TNFRSF21) inhibited the activation of necroptosis of vascular endothelial cells, resulting in significant reduction in transdermal migration of tumor cells, thus controlling tumor metastasis (Wang et al., 2021). IL-8/CXCL8 was found to be regulated by JNK/MAPK8 in colon cancer and became a downstream signal pathway of tumor regrouping induced by necroptosis (Wang et al., 2019). High-risk neuroblastoma (NB) often showed MYCN amplification and decreased susceptibility to the death of programmed cells induced by chemotherapy drugs (Nicolai et al., 2015). Watanabe S. further confirmed that polyphyllin D induced necroptosis in MYCN-amplified NB cells and apoptosis in NB cells without MYCN amplification (Watanabe et al., 2017). TNFR1/TNFRSF1A was a typical necroptosis inducer in pancreatic catheter adenocarcinoma (Seifert et al., 2016). TRAF2 could mediate cross-talk between TNFR1 and TNFR2, affecting signal conduction results of TNF stimulation, including necroptosis (Borghi et al., 2016). HSP90 regulated the stability of MLKL and RIPK3 and was necessary for TNF-stimulated necrosis assembly (Zhao et al., 2016). CHIP/STUB1 regulated necroptosis through ubiquitination and lysosomal-dependent degradation of RIPK1 and RIPK3 (Tang et al., 2018). In addition, RIPK1 in the myeloid progenitor with FLT3 mutations had a strongly increasing tendency (Hillert et al., 2019). Furthermore, we established a nomogram model for predicting the OS of KIRC patients in combination with NRGscore and several clinicopathological characteristics. It showed excellent stability and clinical benefit and was validated in the E-MTAB-1980 cohort.

Due to the strong inflammatory nature of necroptosis, we investigated the correlation between the NRGscore and TIME. Our results indicated that the TME of NRGscore-defined groups was quite distinct. The expression of HLA-related genes had no significant fluctuation, and costimulatory molecules and adhesion molecules were upregulated in the high-risk group. The infiltration level of CD8^+^ T cells that play an antitumor protection role was significantly elevated in the high-risk group. However, patients in the high-risk group had significantly lower OS. In our study, the high-risk group had a significantly elevated immune score and ESTIMATE score, which indicated that the tumor purity of the high-risk group was lower. D Zeng also found that a high immune score was associated with poor prognosis in patients with gastric cancer (Zeng et al., 2018). Similar studies reported that lower tumor purity was related to adverse prognosis and immune escape phenotype (Gong et al., 2020). In addition, as an immunogenic tumor, KIRC could cause immune dysfunction by inducing immunosuppressive cell immersion (Díaz-Montero et al., 2020). We found that Tregs and DCs were highly infiltrated into the TME in patients of the high-risk group. Numerous studies have confirmed that Tregs could form an immunosuppressive microenvironment to promote tumor metastasis and progression (Ohue et al., 2019). In addition, DCs regulated the immune system and induce immune tolerance in a stable state (Audiger et al., 2017). The accumulation of M2 macrophages in the TME was generally associated with poor prognosis (Lan et al., 2019). However, we found that the discovery of M2 macrophages with low components of the TME in KIRC indicated better OS. This contradiction needs further study to be explained.

Somatic mutation is not only the driving factor of tumorigenesis but also TMB can be used as a guiding basis for diagnosis and treatment. As shown in Kaplan–Meier analysis, KIRC patients with high TMB possessed poorer OS survival probability. We also found a significant positive correlation between NRGscore and TMB in KIRC. Some studies have reported that cancer patients with high TMB were more likely to get effective and long-term responses from immunotherapy (Chan et al., 2019; Sholl et al., 2020). Furthermore, we found that NRGscore was significantly positively correlated with the expression of multiple immune checkpoint genes. This means that the immunosuppressive microenvironment played a key role in high-risk patients, who were more likely to benefit from ICIs. We also evaluated the ability of the NRG signature to predict the sensitivity of chemotherapy and targeted drugs in KIRC patients. The results revealed that bortezomib, cisplatin, gefitinib, sunitinib, temsirolimus, and vinblastine had more significant benefits in high-risk patients. We, therefore, believe that NRGscore is helpful for identifying better treatment strategies for individual advanced KIRC patients.

Our research still has limitations. First, this study is a retrospective study in which patient clinical information is prone to bias and requires large, multicenter, prospective studies to further confirm our results. Second, the ability of NRGscore to predict drug efficacy needs to be confirmed by clinical studies with sufficient samples. Finally, the NRGs we included in the study were based on non-KIRC cancer types, and their specific molecular mechanisms for necroptosis in KIRC still need to be further explored.

To sum up, NRGscore can individualize and quantify the necroptosis phenotype of patients and make comprehensive assessments of the clinical, cellular, and molecular characteristics of KIRC patients, including prognosis, clinical characteristics, pathologic stage, histologic grade, TIME, and tumor mutation. NRGscore is an independent prognostic marker for KIRC patients and can be utilized as a guiding indicator in the formulation of treatment strategies for immunotherapy, chemotherapy, and targeted drugs.

## Data Availability

The original contributions presented in the study are included in the article/Supplementary Material, further inquiries can be directed to the corresponding authors.
