# Supplementary material for: Identification and Quantification of Necroptosis Landscape on Therapy and Prognosis in Kidney Renal Clear Cell Carcinoma
Source: Front Genet. 2022 Feb 14;13:832046. doi: 10.3389/fgene.2022.832046 (PMC8882778; doi:10.3389/fgene.2022.832046)
Supplement: Supplementary file 4 [file DataSheet1.DOCX]

Jianguoyun download links:

<https://www.jianguoyun.com/p/DcjJANMQmbqAChi296AE>

The folder where files are located has been marked as described below:

R codes were used in our research

Raw data from public dataset

1. Expression profile and clinical information of TCGA-KIRC cohort
2. Simple nucleotide variation of TCGA-KIRC cohort
3. Expression profile and clinical information of E-MTAB-1980 cohort
4. Attached files required by GSEA, ESTIMATE and CIBERSORT algorithms

Processed data

1. 74 NRGs
2. Expression profile of KIRC samples in TCGA-KIRC cohort (processed with log 2 transformation)
3. Combined files of NRGs expression profile and survival information of KIRC samples in TCGA-KIRC cohort (processed with log 2 transformation) for NMF clustering
4. Combined files of NRGs expression profile and survival information of KIRC samples in TCGA-KIRC and E-MTAB-1980 cohorts (processed with log 2 transformation and corrected background) for prognostic signature construction.
5. Clustering result
6. NRG prognostic signature results (risk score of KIRC patients in TCGA-KIRC and E-MTAB-1980 cohorts, lasso COX regression analysis result, coefficient result)
7. GSEA result of necroptosis-related patterns
8. ESTIMATE results (necroptosis-related patterns and NRG signature)
9. ssGSEA results (immune cells infiltration and functions in necroptosis-related patterns; immune functions in NRG signature)
10. GO and KEGG pathways results of DEGs between necroptosis-related patterns
11. Nomogram results of train and test sets
12. GSEA results
13. CIBERSORT result
14. Somatic mutation (TMB and preparation file for waterfall plots)
